# Construction of a non-contact community treatment centre for asymptomatic and mildly symptomatic COVID-19 patients during the COVID-19 pandemic

**DOI:** 10.1017/S0950268821000996

**Published:** 2021-04-27

**Authors:** Si Hyun Kim, Youn Jeong Kim, Yeon Jeong Jeong, Ji Hye Park, Shin Young Lee, Mi Sun Choi, Sang Yong Kim

**Affiliations:** 1 Division of Infectious Disease, Department of Internal Medicine, College of Medicine, The Catholic University of Korea, Seoul, South Korea; 2 Department of Internal Medicine, Incheon St. Mary's Hospital, College of Medicine, The Catholic University of Korea, Incheon, South Korea; 3 Infection Control Team, Incheon St. Mary's Hospital, College of Medicine, The Catholic University of Korea, Incheon, South Korea; 4 Department of Pediatrics, Incheon St. Mary’s Hospital, College of Medicine, The Catholic University of Korea, Seoul, South Korea

**Keywords:** COVID-19, infection prevention, SARS-CoV-2

## Abstract

The explosive outbreak of COVID-19 led to a shortage of medical resources, including isolation rooms in hospitals, healthcare workers (HCWs) and personal protective equipment. Here, we constructed a new model, non-contact community treatment centres to monitor and quarantine asymptomatic and mildly symptomatic COVID-19 patients who recorded their own vital signs using a smartphone application. This new model in Korea is useful to overcome shortages of medical resources and to minimise the risk of infection transmission to HCWs.

Coronavirus disease 2019 (COVID-19) has rapidly spread globally since the novel severe acute respiratory syndrome coronavirus 2 was first reported in Wuhan, China at the end of 2019 [1]. In South Korea, the number of COVID-19 patients increased rapidly especially in specific religious facilities in Daegu City and Gyeongbuk regions in February 2020 during the early stages of the pandemic [2, 3]. The explosive outbreak of COVID-19 led to a shortage of medical resources, including isolation rooms in hospitals, healthcare workers (HCWs) and personal protective equipment (PPE). In March 2020, community treatment centres (CTCs) were designed for quarantining and monitoring asymptomatic and mildly symptomatic patients with laboratory-confirmed COVID-19 by the South Korean government [4, 5]. To improve clinical outcomes and efficiently allocate medical resources, the regional COVID-19 management team assigned patients to care sites depending on disease severity and site availability during the outbreak in Daegu [5].

Incheon Metropolitan City is a city located in northwestern South Korea that borders Seoul and Gyeonggi to the east and has a population of approximately 3 million. It had 2757 confirmed cumulative cases of COVID-19, including 25 deaths, as of 29 December 2020. Here, we report our experience of a CTC in Incheon, including its structure and protocols that is operated by Incheon St. Mary's Hospital, a teaching hospital, based on a non-contact method and patients' clinical characteristics. Statistical analyses were performed in SPSS (version 14.0; SPSS Inc., Chicago, IL, USA). Continuous data are expressed as the median (range), and categorical data are expressed as percentages. The *χ*^2^ test or Fisher's exact test was used for categorical variables and Student's *t* test or the Mann–Whitney *U* test for continuous variables.

The CTC in Yeongjong-do, Incheon, which was converted from a sports centre to 59 rooms, can accommodate up to 118 patients by using it as a double room when the number of confirmed cases surge, and opened on 9 September 2020. The patient area (dirty zone) was physically separated from the monitoring area (clean zone) where HCWs and operating staff stayed (Fig. 1a). To minimise the transmission risk through contact with or respiratory droplets expelled by COVID-19 patients, we constructed the patient care and polymerase chain reaction testing rooms such that patients and HCWs did not come into direct contact based on our previous experience [6].

**Fig. 1.**
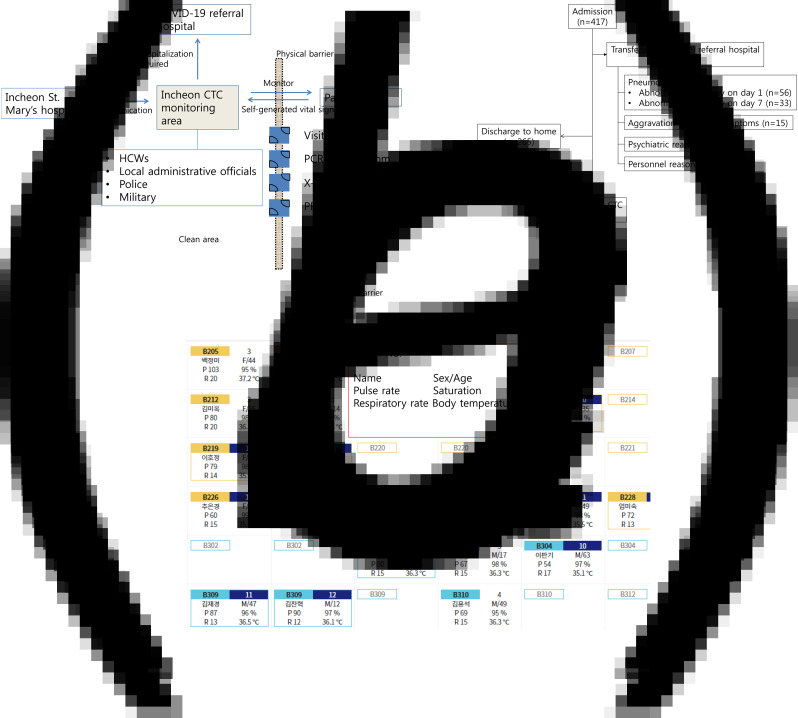
Overall structure of the CTC located in Yeongjong-Do, Incheon. (a) Diagram of the CTC. (b) Schematic diagram of patient outcomes in the CTC. (c) Monitor screen showing patients' self-generated vital signs using a smartphone application.

Two physicians, six nurses and one radiologic technician currently work at the CTC. Personnel from the local government, military and police provided various services. Common drugs such as antitussives, antipyretics, digestives, antihistamines and emergency drugs were prepared. Asymptomatic or mildly symptomatic COVID-19 patients were admitted to the CTC if they did not meet the following Korea Centers for Disease Control and Prevention (KCDC) criteria for high-risk groups: (1) ⩾65 years of age, and (2) any of the underlying conditions such as chronic liver disease, heart failure, chronic renal disease, pulmonary disease, diabetes mellitus, malignancy, HIV and use of immunosuppressant [7].

Each room of the CTC was equipped with an automatic blood pressure monitor, digital thermometer and pulse oximeter. Admitted patients were asked about their symptoms and measured their own vital signs, including body temperature, pulse rate, blood pressure and oxygen saturation, twice per day and recorded the results using a smartphone application. HCWs at the CTC checked their responses and conducted telephone consultations. If patients required specialists such as psychiatrists, physicians at the CTC could communicate with specialists at Incheon St. Mary's Hospital, and specialists sometimes conducted telephone consultations.

All patients underwent routine chest X-rays on days 1 and 7. These were checked by a radiologist at Incheon St. Mary's Hospital through the electronic medical record system and the findings were shared with HCWs at the CTC. If patients had persistent fever or symptoms consistent with pneumonia, they underwent an additional chest X-ray. In the case of an emergency, HCWs on duty entered the patient area wearing PPE. If patients required hospitalisation or developed pneumonia, they were immediately transferred to a COVID-19 referral hospital with a negative-pressure isolation room. Patients were discharged if they meet the clinical or testing criteria based on the KCDC [7].

Infectious disease specialists and an infection prevention team taught HCWs and operating staff about infection control and how to put on PPE at their first visit and once per month thereafter.

Up to 31 December 2020, a total of 417 patients (215 males and 202 females) with a median age of 40 years (interquartile range (IQR), 29–52 years) had been admitted to the CTC. Forty-one cases remained at the CTC, whereas 111 patients (26.6%) were transferred to a COVID-19 referral hospital (Fig. 1b). The most common reason for transfer was pneumonia (*n* = 89), followed by aggravating symptoms such as persistent fever or dyspnoea (*n* = 15), psychiatric reasons (*n* = 4) and personnel reasons (*n* = 3). Fifty-six (13.7%) of 410 patients who underwent a chest X-ray on day 1 had pneumonia, whereas 33 (10.2%) of 334 patients who did not initially have pneumonia subsequently developed pneumonia that was detected by a second chest X-ray on day 7. Table 1 compares the 34 patients with pneumonia on hospital day 7 who did not have pneumonia on hospital day 1 and 297 patients without pneumonia. Patients who developed pneumonia were older (median 36 *vs.* 45 years old, *P* = 0.02) and had a higher body mass index (median 24 *vs.* 35, *P* = 0.02) (Table 1).

**Table 1.** Comparison between the patients with and without pneumonia on hospital day 7

Characteristics	Normal (*n* = 297)	Pneumonia (*n* = 34)	*P* value
Age, years, median (IQR)	36 (27–49)	45 (35.3–54)	0.02
Sex, male	157 (52.9%)	18 (52.9%)	0.79
BMI, kg/m^2^, median (IQR)	24 (21–26)	25 (22.8–27)	0.02
Current smoker	40 (13.5%)	5 (14.7%)	0.79
Asymptomatic	55 (18.5%)	4 (11.8%)	0.48
Underlying disease			
Hypertension	13 (4.4%)	3 (8.8%)	0.22
Diabetes mellitus	2 (0.7%)	0 (0%)	1.0
Dyslipidaemia	3 (1%)	1 (2.9%)	0.35
Asthma	3(10.0%)	0 (0%)	1.0
Initial heart rate, bpm	86 (79–96)	85.5 (80–98.5)	0.83
Initial respiratory rate, times/min	15 (14–17)	16 (14–19)	0.07
Initial saturation, %	98 (97–98)	97 (96.8–98)	0.73
Length from symptom onset to diagnosis, days (IQR)	2 (1–4)	2 (0.8–4)	0.56

We constructed a non-contact CTC to monitor and quarantine asymptomatic and mildly symptomatic COVID-19 patients who recorded their own vital signs using a smartphone application. This new model in Korea is useful to overcome shortages of medical resources and to minimise the risk of infection transmission to HCWs.

## Data Availability

The datasets used during the current study are available from the corresponding author on a reasonable request.
